# Neuron-tumor interplay in colorectal cancer: from mechanisms of onset and progression to targeted therapies

**DOI:** 10.1186/s12964-026-02938-5

**Published:** 2026-05-16

**Authors:** Zhenni ChenLiu, Dong Tang

**Affiliations:** 1 Clinical Medical College, Yangzhou University, Yangzhou, Jiangsu Province 225000 China; 2 Department of General Surgery, Institute of General Surgery, Northern Jiangsu People’s Hospital Affiliated to Yangzhou University, Northern Jiangsu People’s Hospital, The Yangzhou Clinical Medical College of Xuzhou Medical University, The Yangzhou School of Clinical Medicine of Dalian Medical University, The Yangzhou School of Clinical Medicine of Nanjing Medical University, Northern Jiangsu People’s Hospital, Clinical Teaching Hospital of Medical School, Nanjing University, Yangzhou, 225000 China

**Keywords:** Nervous system, Colorectal cancer, Enteric nervous system, Immune response, Targeted therapy

## Abstract

Colorectal cancer (CRC) is a major cause of cancer-related mortality worldwide, traditionally attributed to genetic mutations and epigenetic alterations. However, with the emergence of cancer neuroscience, a paradigm shift has occurred, revealing the central role of the nervous system in the initiation, progression, and metastasis of CRC. The gastrointestinal tract, being the only organ in the body with an independent peripheral nervous system — the enteric nervous system (ENS) — provides a unique anatomical and functional basis for neuro-tumor interactions. This review systematically explores the mechanisms by which the nervous system regulates CRC cell proliferation, invasion, and immune microenvironment remodeling through neurotransmitters (such as norepinephrine and acetylcholine) and neurotrophic factors (including NGF and BDNF). We also delve into the role of the brain-gut-microbiome axis, particularly its influence on tumor progression through metabolic reprogramming and neuroimmune crosstalk. Finally, we discuss novel therapeutic strategies based on the bidirectional communication between the nervous system and CRC, offering new perspectives for precise interventions in CRC.

**Graphical Abstract:**

**Figure:**
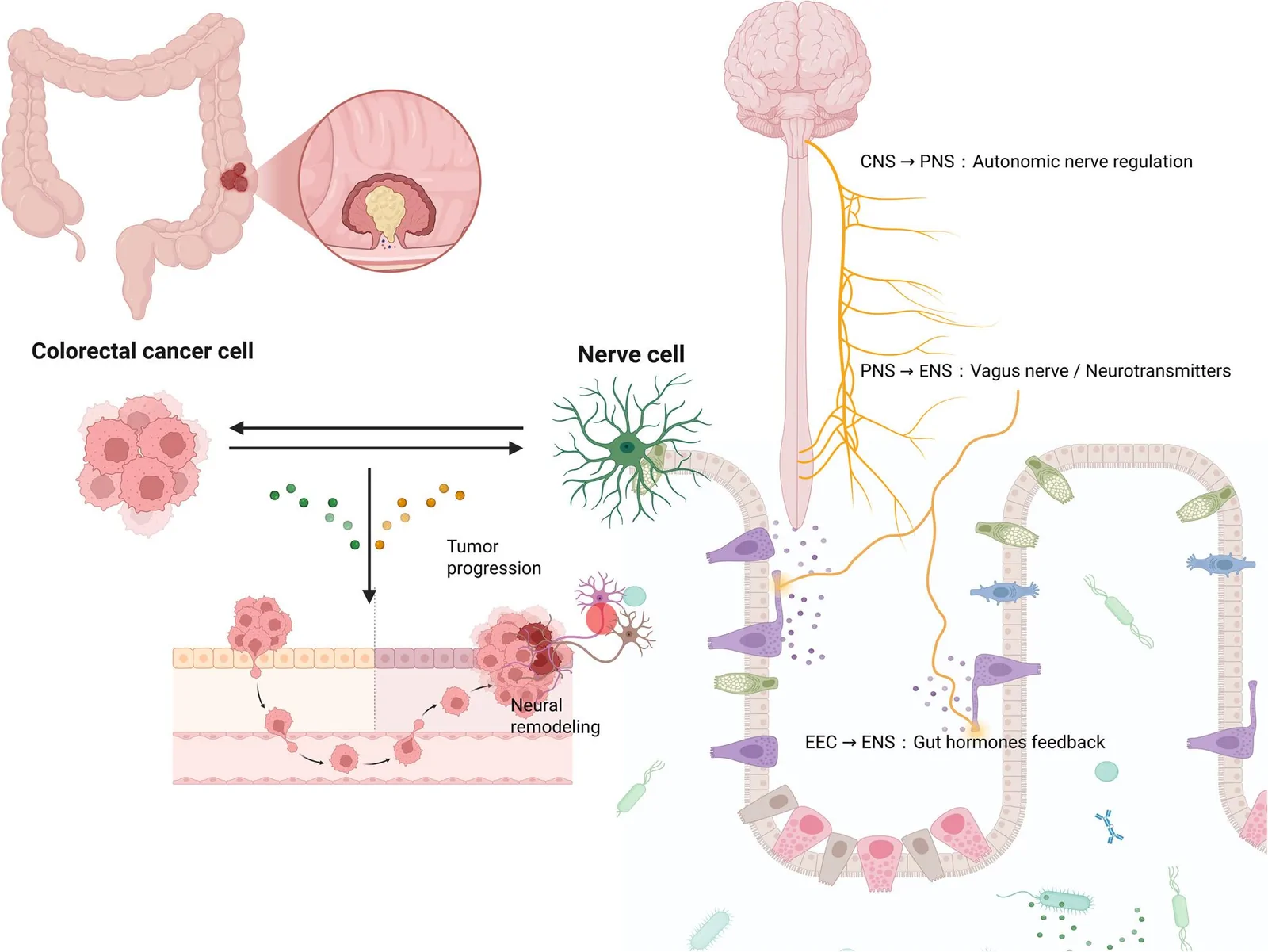

## Introduction

Colorectal cancer (CRC), as the third most common malignancy globally, continues to have high incidence and mortality rates, posing a significant public health challenge. Although significant progress has been made in the fields of molecular targeted therapy and immunotherapy in recent years, tumor resistance and recurrence with metastasis remain major obstacles in clinical treatment. For a long time, it has been widely accepted in the academic community that the development of CRC is mainly driven by the synergistic effects of genetic mutations and epigenetic dysregulation. Mutations in key genes such as adenomatous polyposis coli (APC), Kirsten rat sarcoma viral oncogene homolog (KRAS), and tumor protein p53 (TP53) have been identified as core molecular events in the progression of CRC [1]. However, perineural invasion (PNI) is identified in certain patients upon diagnosis, a factor strongly correlated with adverse prognostic outcomes [2]. Consequently, beyond genetic mutations, the nervous system—serving as the body’s most extensive and efficient informational network—may exert a dominant regulatory influence on CRC biology. These insights challenge the conventional perspective of "tumors as isolated entities dependent exclusively on autonomous proliferative advantages," unveiling CRC as a sophisticated pathological condition characterized by considerable "environmental adaptability and regulatory complexity." It can actively recruit, reshape, and utilize the nervous system, turning it into an accomplice to serve its own malignant progression.

In recent years, investigations into the interplay between neural processes and tumors have emerged as a critical frontier within CRC research. Accumulating evidence indicates that malignancies can actively co-opt elements of the nervous system—such as neurotransmitters, neurotrophic factors, and neuroendocrine pathways—to facilitate tumor advancement and escape from immune surveillance. The central nervous system (CNS) regulates the systemic immune state through the HPA axis and the sympathetic-adrenal medullary axis (SAM) system; while the peripheral and enteric nervous system (ENS) directly shapes the tumor microenvironment (TME) by releasing mediators such as norepinephrine (NE), acetylcholine (ACh). NGF, and BDNF [3]. Especially in CRC, the ENS forms a dynamic bidirectional network with the CNS through the vagus nerve and sympathetic pathways, highlighting its unique position. The establishment of this "neuro-immune-tumor" ternary interaction framework marks a shift in the research paradigm of CRC from the traditional tumor-centric view to an integrated microenvironmental systems view with neuroregulation. Against this background, this review aims to systematically dissect the multifaceted functions of neural signaling in the occurrence and development of CRC: from the mechanisms of neuro-dependent tumor initiation and progression to tumor-induced neural remodeling phenomena, and finally to look forward to innovative therapeutic strategies targeting neuro-tumor dialogue [4]. By reviewing the latest findings in interactive mechanisms and clinical translation progress, this review aims to provide theoretical basis and direction for the development of precision therapies targeting the neural dimension of CRC.

## The bidirectional communication between the nervous system and colorectal cancer

### Neural functional network in colorectal cancer

**Fig. 1:**
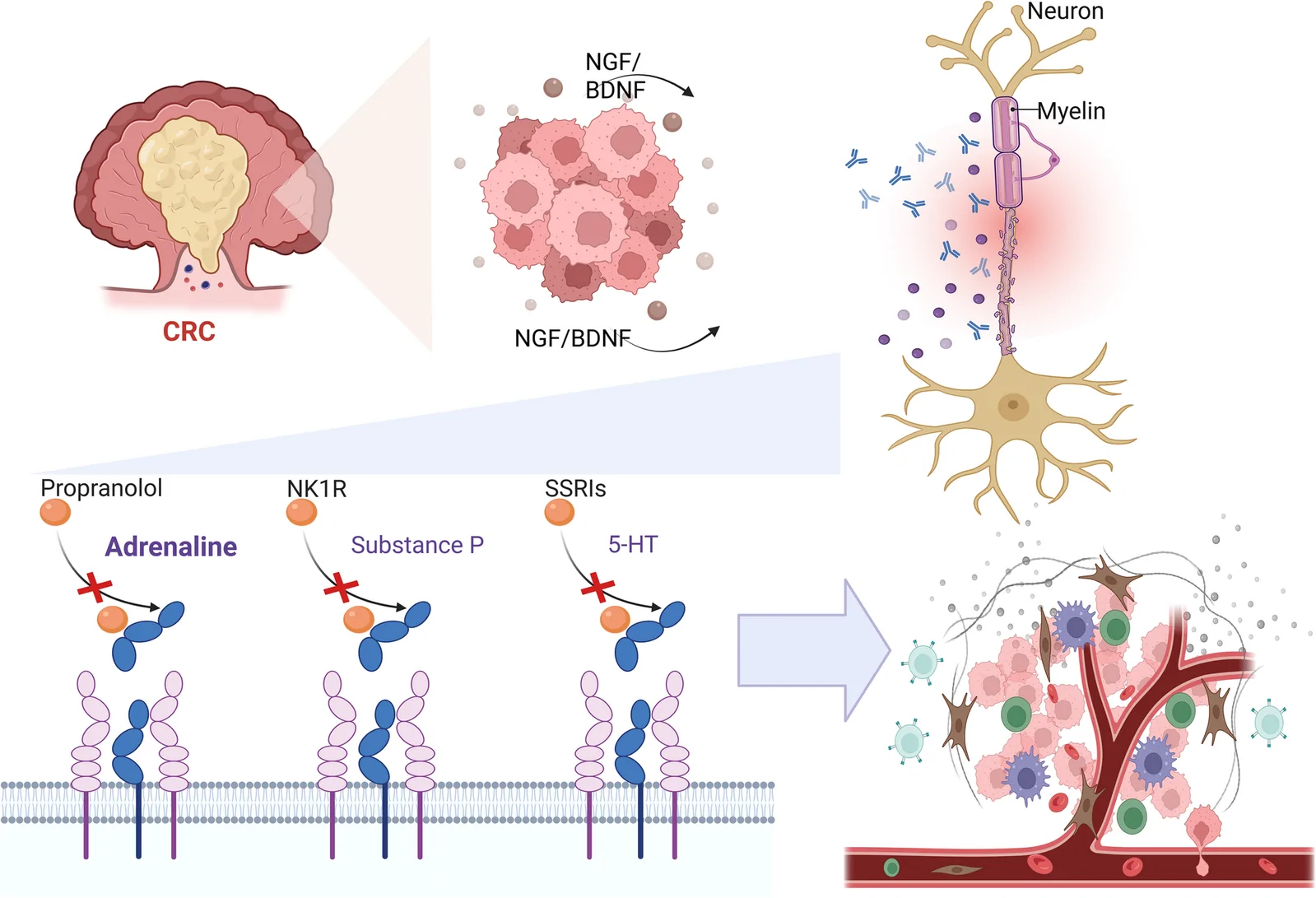
The neural functional network and response strategies in CRC. CRC cells can actively "hijack" neural signaling mechanisms by secreting various factors, including nerve growth factor (NGF) and brain-derived neurotrophic factor (BDNF), to induce the regeneration and infiltration of tumor-associated nerves. Blocking β-adrenergic receptors with propranolol, inhibiting substance P signaling with NK1R antagonists, and reducing serotonin (5-HT) activity with SSRIs can effectively reverse the aforementioned neuro-immune suppressive effects, thereby enhancing anti-tumor immune responses

The peripheral nervous system, enteric nervous system, and central nervous system hierarchically regulate the gastrointestinal tract, which serves as a typical peripheral organ. A growing body of evidence points to the nervous system as a key driver of CRC initiation, progression, and metastasis. CRC cells are capable of actively "hijacking" neural signaling pathways through the secretion of neurotrophic factors including nerve growth factor (NGF) and brain-derived neurotrophic factor (BDNF), a process that induces tumor-associated neurogenesis. Concurrently, dynamic modulation of tumor cell behavior and TME is achieved by multi-tiered neural networks via the release of neurotransmitters and neuropeptides. The malignant phenotype of CRC is ultimately shaped by this sustained bidirectional crosstalk arising from these interconnected processes [5]. Within this framework, tumor-associated neurogenesis has emerged as a key mechanism linking the nervous system to tumor progression (Fig. 1). Through neurotransmitter and neuroendocrine signaling, the nervous system orchestrates the immune microenvironment, forming an integrated “neuro–immune–tumor” axis that collectively promotes CRC development and dissemination [6]. Tumor-derived neurotrophic factors activate downstream signaling pathways by binding to receptors like tropomyosin receptor kinase A (TrkA) and TrkB. This activation promotes neuronal proliferation, differentiation, and survival, ultimately leading to the formation of structurally and functionally active neural networks within the tumor microenvironment [7]. Tumor-derived Jagged2 (JAG2) has been shown to promote macrophage polarization toward an immunosuppressive state through activation of NOTCH3 signaling. These reprogrammed macrophages exhibit increased neurotrophic and chemotactic capacities, facilitating tumor cell migration and neurite extension, and thereby reinforcing a positive feedback circuit within the neuro–tumor–immune axis [8]. Importantly, tumor-associated neurogenesis extends beyond neuronal expansion to encompass the establishment of functional signaling hubs. These hubs secrete norepinephrine, acetylcholine, and a variety of neuropeptides, providing sustained proliferative and migratory cues to tumor cells while concurrently reshaping the immune microenvironment [9]. In parallel, neural remodeling is often accompanied by increased tumor cell plasticity, among which neuroendocrine differentiation (NED) represents a critical phenotype. CRC cells undergoing NED acquire enteroendocrine cell (EEC)-like secretory functions, producing neuroendocrine mediators such as serotonin (5-HT) and peptide YY (PYY). These factors not only act in an autocrine manner on tumor cells but also engage surrounding neurons and immune cells via paracrine and neuron-like signaling, thereby amplifying neuro–tumor interactions and promoting invasive behavior and immune evasion [10].

#### The nervous system as a pro-tumorigenic messenger

When the sympathetic nervous system (SNS) is activated, the NE and epinephrine (Epi) released from its terminals bind to the β2-adrenergic receptors (β2-AR) on the surface of tumor and immune cells, triggering the transcriptional activity of downstream target genes. Sympathetic nerve activity inhibits the secretion of intestinal endocrine hormones and exacerbates the progression of inflammatory bowel disease (IBD), irritable bowel syndrome (IBS), and CRC [11]. In mouse models, β-adrenergic signaling inhibits the effector phenotype in CD8 T cells and disrupts checkpoint inhibitor therapy [12]. Activation of β2-adrenergic receptors (β2-AR) impairs dendritic cell (DC) function, as evidenced by reduced antigen-presenting capacity and decreased expression of co-stimulatory molecules such as CD80 and CD86. At the same time, it promotes the expansion of regulatory T cells (Tregs), collectively fostering an immunosuppressive microenvironment [13]. NE further engages a tumor cell–intrinsic β2-AR–CREB–c-Myc signaling cascade, leading to the activation of the RAS–MAPK and PI3K–AKT oncogenic pathways. In parallel, NE reshapes TME in a STAT3-dependent manner by driving M2-like polarization of tumor-associated macrophages (TAMs), expanding myeloid-derived suppressor cells (MDSCs), and inducing CD8⁺ T cell exhaustion. These coordinated effects synergistically facilitate tumor immune evasion [14]. Notably, the α2-adrenergic receptor (α2-AR) exhibits the opposite anti-tumor effect compared to β2-AR, and this effect is entirely dependent on the host immune system: in immunocompetent mice (including immune checkpoint blockade-resistant tumor models), α2-AR agonists alone can significantly inhibit tumor growth; however, this effect completely disappears in immunodeficient mice or Adra2a gene knockout mice. Moreover, in immunodeficient mice reconstituted with human peripheral blood lymphocytes, α2-AR agonists still show significant efficacy against human CRC xenografts, and this effect can be reversed by α2-AR antagonists [15].

As the core component of the parasympathetic system, the vagus nerve (VN) uses its parasympathetic efferent fibers to establish a “vagus-ENS loop” together with cholinergic neurons residing in ENS. Through this loop, ACh is rhythmically released, helping to preserve both the integrity of the intestinal epithelial barrier and immune homeostasis. By engaging the cholinergic anti-inflammatory pathway (CAP), neuroendocrine-immune circuits, and the microbiota-vagus-immune axis, this loop regulates various TME components—including the gut microbiota and cancer stem cells—thereby influencing CRC progression [16]. In terms of immune regulation, NE promotes the polarization of TAMs towards the M2 type and inhibits the maturation of dendritic cells, thereby weakening antigen-presenting functions and impairing the initiation of anti-tumor immune responses. In contrast, ACh released by the parasympathetic nervous system in a breast cancer mouse model increases the proportion of CD8⁺ T cells and natural killer (NK) cells, as well as the levels of perforin and granzyme B (GZMB). To some extent, the parasympathetic nervous system may be a potential target for immune regulation in CRC [17].

In the CRC microenvironment, ACh is primarily synthesized via two distinct mechanisms: secretion by cholinergic neurons within the ENS, and a non-neuronal autocrine pathway mediated by the upregulation of choline acetyltransferase (ChAT) and the vesicular ACh transporter (VAChT) in CRC cells. Research has demonstrated that CRC cells exhibit high expression of the M3 muscarinic receptor (M3R). Through M3R activation, ACh induces transactivation of the ERK/MMP-7 signaling axis, thereby enhancing the production of matrix metalloproteinases and facilitating tumor invasion and metastatic dissemination [18]. Genetic deletion or pharmacological inhibition of M3R markedly attenuates nuclear translocation of β-catenin and suppresses the expression of Wnt target genes, leading to effective restraint of tumor progression [19]. The ACh-M3R axis can induce NGF expression and secretion to activate the TRK receptor in cancer cells, continuously amplifying WNT and YAP signals to drive proliferation, while recruiting and reshaping cholinergic nerve terminals through a paracrine loop gradient, increasing local ACh bioavailability, and forming a "neuro-tumor" positive feedback loop [20]. Additionally, it has been reported that high expression of the α7 nicotinic ACh receptor (α7nAChR) in TAMs in CRC is associated with a low incidence of liver metastasis, which is related to the inhibition of metastasis by α7nAChR through the JAK2/STAT3 signaling pathway [21]. α7nAChR is also an important component of the potential mechanism regulating the anti-inflammatory effects of cholinergic neurons. Therefore, the interaction of ACh receptors in CRC collectively amplifies tumor-promoting signaling in cancer cells, including the activation of WNT and YAP pathways, becoming an accomplice in tumor progression [22].

The cell bodies of sensory nerves are aggregated in the dorsal roots of spinal nerves or cranial nerve ganglia (pseudounipolar neurons), with their peripheral branches terminating in the intestinal mucosa, directly participating in the regulation of the CRC microenvironment. Tumor-innervating nociceptive neurons significantly contribute to immunosuppression through specific molecular mechanisms: reactive oxygen species generated by CRC cells stimulate the activation of NGF, which subsequently prompts these neurons to release calcitonin gene-related peptide (CGRP). Released CGRP binds to corresponding receptors on CRC cells, facilitating tumor progression and protective autophagy, while simultaneously engaging with receptors on CD8⁺ T cells to induce functional exhaustion of effector T cells [23]. At the mechanistic level, the CGRP–RAMP1 signaling pathway selectively suppresses the secretion of Th1-type cytokines, including IFN-γ and IL-12, as well as impedes dendritic cell maturation. Concurrently, it enhances the proliferation of regulatory T cells, collectively establishing a profoundly immunosuppressive tumor microenvironment [24]. Additionally, in a mouse breast tumor model, substance P from sensory nerves, which can detach from pain regulation functions, binds to the tachykinin receptor TACR1 to activate cancer growth and metastasis-related genes, enhancing the metastatic capacity of tumors [25]. This suggests that the reprogramming of sensory neurotransmitter functions may be an important inducer of CRC metastasis.

#### The triangular interaction between the ENS, gut microbiota, and CRC

The ENS acts as a "local neural command center" for the interaction between gut microbiota and the host. Its function is regulated in real-time by the CNS and primarily operates through the submucosal plexus (which secretes ACh and vasoactive intestinal peptide) and the myenteric plexus (which has three subtypes). In addition to neurons, the supportive network formed by enteric glial cells (EGCs) is also a key participant in neuro-tumor interactions. The ENS can dynamically interact with TME by regulating neurotransmitters, neuropeptides, gut microbiota, and neurotrophic factors [26]. The colorectum is the main colonization site for gastrointestinal bacteria. CRC cells can reshape the gut microbiota ecology (e.g., by enriching Fusobacterium nucleatum) to form a pro-tumorigenic community and feedback-regulate neural plasticity through neurotrophic factors [27]. For example, CRC-associated pathogens such as Fusobacterium nucleatum, Porphyromonas gingivalis, and Pseudomonas anaerobic bacteria can precisely hijack the Wnt/β-catenin, MAPK, and NF-κB pathways through various adhesion-receptor interactions. These interactions not only drive autonomous proliferation of CRC cells but also reshape the immune-stromal microenvironment [28]. Specifically, Fusobacterium nucleatum (F.nucleatum) activates Wnt/β-catenin through the FadA toxin, while Porphyromonas gingivalis drives an inflammatory microenvironment through TLR/NF-κB, both promoting the self-renewal of cancer stem cells (CSCs) [29, 30]. Interestingly, neurotrophic mediators such as NGF and BDNF can also engage receptor tyrosine kinase (RTK)-dependent oncogenic signaling and drive the transformation of CSCs [31]. In the CRC microenvironment, NGF is mainly enriched in the stroma-3 fibroblast subpopulation (CAF-S3), with expression levels significantly higher than in normal tissue. NGF induces sympathetic nerve axons to extend directionally into the tumor stroma, increasing nerve density, and thereby promotes tumor proliferation through YAP-dependent mechanotransduction signaling [32]. Members of the neurotrophic factor family promote axonogenesis and nerve survival through tropomyosin receptor kinases (Trk). In CRC, overexpressed NGF engages its high-affinity receptor TrkA, triggering activation of the MAPK/ERK signaling cascade and upregulation of matrix metalloproteinases, thereby promoting tumor cell proliferation, invasion, and perineural dissemination. Additionally, NGF can inhibit NK cells from secreting IFN-γ and weaken NK cell cytotoxicity and anti-tumor immunity [33]. Chemotherapy-resistant CRC cells can enhance migratory activity by upregulating TrkB/C through the PI3K-AKT-mTOR pathway [34]. Therefore, NGF is not only a biomarker of stromal activation in human CRC but also a key molecule driving YAP/AKT pathway activation and tumor progression through neuro-stromal interactions. CRC cells can also secrete BDNF to activate local neuronal synapses, not only accelerating tumor proliferation and highlighting the neurodevelopmental dependence of CRC but also inducing CD8⁺ T cell functional exhaustion through the β-AR signaling pathway, forming an immune-evasive microenvironment [15]. Thus, the interaction between the gut microbiota, ENS, CRC stem cells, and the TME affects the development and progression of CRC, influencing immune responses, metabolic pathways, and even genetic stability within the tumor [35].

EGCs and Schwann cells (SCs) both originate from the neural crest (NC) and migrate along the vagus nerve to the gut during embryogenesis, differentiating into ENS astrocyte-like cells. Recent studies have confirmed that EGCs play a key role in CRC progression, metastasis, and immune evasion by secreting glial cell-derived neurotrophic factor and its downstream signaling network. Glial cell-derived neurotrophic factor (GDNF) activates the SRC-HER2 and AKT signaling pathways by binding to the RET receptor tyrosine kinase, promoting tumor cell proliferation, migration, and invasion [36]. Epigenetic studies have shown that demethylation of the GDNF family receptor α1 (GFRA1) promoter can enhance AKT phosphorylation and upregulate c-Jun expression, thereby inducing epithelial-mesenchymal transition (EMT) and accelerating the invasive growth of CRC [37]. In addition, studies have shown that glial cell-derived neurotrophic factor can induce tumor cells to secrete CX3CL1. The CX3CL1-CX3CR1 signaling drives TCF7⁺ regulatory T cells to transform into a more immunosuppressive TNFRSF9⁺ Treg phenotype, enhancing their metabolic competitive ability and forming a metabolic immunosuppressive microenvironment. Moreover, this axis mediates the enrichment of M2-type macrophages in the pre-metastatic niche, with their infiltration levels significantly negatively correlated with CD8⁺ T cells, indicating their central role in CRC immune evasion [38, 39].

Neuromedin U (NMU) is a highly conserved neuropeptide that signals through two principal G protein–coupled receptors, NMUR1 and NMUR2 (Table 1). Accumulating evidence indicates that NMU and its receptors are aberrantly expressed across multiple tumor types and participate in several critical steps of tumor initiation and progression. In colorectal cancer (CRC), NMUR1 and NMUR2 exhibit marked functional divergence, suggesting subtype-specific regulatory roles of this signaling axis in tumor biology [40]. Mechanistically, the NMU–NMUR1/NMUR2 axis constitutes a neuro–tumor signaling pathway with considerable pharmacological potential. Non-canonical Hippo pathway activation by exogenous NMU leads to the recruitment of transcriptional co-activators YAP/TAZ, which potently stimulates CRC cell proliferation, migration, and invasion while also inducing resistance to radiotherapy [41]. At the level of receptor subtypes, clinical correlation analyses have revealed a significant association between elevated NMUR1 expression and poorer overall survival, pointing to NMUR1 as a potential prognostic biomarker in CRC. Importantly, work by Zhou and colleagues demonstrated that NMU impairs immune surveillance by suppressing CD8⁺ T cell–mediated antitumor activity via an NMUR1-dependent mechanism [42]. This observation reinforces the critical involvement of NMUR1 in immune regulation and raises the possibility of using it to predict immunotherapy responses. By contrast, NMUR2 appears to be more involved in controlling tumor cell–intrinsic invasive phenotypes. When NMUR2-positive CRC cells are exposed to NMU, a Ca^2^⁺–ERK signaling cascade is activated in an NMUR2-dependent manner, leading to upregulated integrin expression and enhanced cell adhesion and motility, thereby significantly boosting their migratory and invasive capacities [43]. Collectively, these findings indicate that NMUR2 primarily governs pro-invasive programs within tumor cells rather than modulating the tumor microenvironment.

**Table 1:** Functional divergence of NMUR1 and NMUR2 in colorectal cancer

Dimension	NMUR1	NMUR2
Primary Localization	Immune cells/TME (Tumor Microenvironment)	CRC tumor cells
Expression Trend	Decreased or immune-related	Upregulated
Core Pathway	Immune checkpoint-related	Ca^2^⁺–ERK
Main Function	Regulates immune response	Promotes invasion and metastasis
Impact on Tumor	Suppresses malignancy or modulates immunity	Clearly pro-tumorigenic
Clinical Significance	Biomarker for immunotherapy	Biomarker for metastasis and prognosis

5-HT, a monoamine inhibitory neurotransmitter, is predominantly produced in the gastrointestinal tract: approximately 95% is synthesized by enterochromaffin (EC) cells and enteric serotonergic neurons, whereas only ~ 5% originates from CNS. Clinical studies have shown that elevated plasma 5-HT levels in patients with CRC are significantly associated with increased risk of recurrence and poor prognosis, suggesting its potential utility as a prognostic biomarker. Mechanistically, neuron-derived 5-HT regulates the Wnt/β-catenin signaling pathway via HTR1B/1D/1F receptors, thereby maintaining cancer stem cell stemness and promoting tumor initiation [44]. Peripheral 5-HT, in contrast, drives tumor progression primarily through HTR2B-mediated signaling. Activation of HTR2B stimulates the ERK/MAPK and TGF-β pathways, leading to enhanced CRC cell proliferation, epithelial–mesenchymal transition (EMT), and metastatic dissemination [45]. Notably, pharmacological inhibition of HTR2B using small-molecule antagonists (e.g., GM-60186) significantly downregulates dual-specificity phosphatase 4 (DUSP4), upregulates the pro-apoptotic factor BID, and blocks TGF-β–induced EMT, resulting in marked antitumor efficacy in human CRC xenograft models [45]. Beyond canonical receptor-dependent mechanisms, 5-HT also exerts epigenetic regulatory functions. Through the transporter SLC22A3 and transglutaminase 2 (TGM2), 5-HT mediates serotonylation of histone H3 at glutamine 5 (H3Q5ser), thereby reprogramming the phenotype of cancer-associated fibroblasts (CAFs) within the tumor microenvironment. This modification not only promotes CAF activation but also induces M2 macrophage polarization, collectively enhancing tumor invasiveness [46].

#### Dynamic interplay between enteroendocrine cells and the enteric nervous system in colorectal cancer

During CRC development, a subset of tumors undergoes neuroendocrine differentiation (NED), acquiring features reminiscent of EECs, including the expression of markers such as chromogranin A and synaptophysin, as well as the ability to secrete neurotransmitters and peptide hormones. This phenotypic transition confers enhanced adaptive fitness and survival capacity to tumor cells [47]. NED constitutes a notable phenotypic transition in CRC, frequently observed in adenocarcinomas and dedifferentiated tumors, and is generally considered an indicator of disease progression [48]. CRC cells can secrete a diverse array of enteroendocrine hormones, including glucagon-like peptide-1 (GLP-1), PYY, and somatostatin. Beyond their roles in intestinal metabolic regulation, these hormones influence tumor cell behavior and shape local immune responses via both autocrine and paracrine signaling [49]. Importantly, the presence of neuroendocrine carcinoma components in CRC is closely linked to unfavorable clinical outcomes, highlighting the independent adverse prognostic relevance of NED [50].

EECs are highly specialized sensory cells within the intestinal epithelium that detect luminal nutrients, metabolites, and microbial signals, translating them into neural and endocrine outputs. As central integrators of luminal cues, EECs establish a tightly coupled functional network with ENS through synapse-like structures as well as paracrine and endocrine signaling, thereby forming a localized “neuro–endocrine–immune” regulatory unit. This cross-hierarchical axis plays a fundamental role in maintaining intestinal homeostasis, barrier integrity, and immune balance, and has attracted increasing attention in the context of CRC pathogenesis [51]. Mechanistically, the ENS fine-tunes the secretory profile and release dynamics of EEC-derived hormones through classical neurotransmitters, including acetylcholine, norepinephrine, and substance P. Conversely, EEC-derived incretin hormones—such as GLP-1, GLP-2, and PYY—act on G protein–coupled receptors (GPCRs) to feedback-regulate ENS neuronal excitability and firing patterns, thereby influencing gut motility, epithelial barrier function, and local immune states [52]. For instance, GLP-2 binds to GLP-2 receptors (GLP-2R) expressed on enteric neurons, activating neural circuits that promote the release of vasoactive intestinal peptide (VIP) and nitric oxide (NO), which in turn regulate epithelial SGLT1 expression, ion transport, and barrier integrity. Genetic ablation of GLP-2R abolishes GLP-2–induced enhancement of intestinal absorption, while pharmacological inhibition of ENS activity similarly negates its biological effects, highlighting the indispensable role of the EEC–ENS axis in this process [53]. In contrast, the GLP-1–ENS axis predominantly regulates neuronal excitability and intestinal motility. GLP-1 receptors are broadly expressed in specific ENS neuronal subsets, and their activation enhances neuronal firing frequency, thereby modulating peristaltic rhythms and secretory functions. Moreover, GLP-1 signaling contributes to the integration and transmission of vagal afferent inputs via the gut–brain axis. Importantly, GLP-1 receptor (GLP-1R) signaling may also influence antitumor immunity by reshaping neuro–immune interactions. For example, GLP-1R antagonism has been shown to enhance T cell–mediated antitumor responses, suggesting a functional role for this pathway in regulating the CRC immune microenvironment [54]. Recent large-scale epidemiological evidence suggests that the use of GLP-1 receptor agonists (GLP-1RAs) correlates with a lower risk of colorectal cancer in individuals with type 2 diabetes, pointing to a previously overlooked connection between metabolic signaling and cancer development. As a key hormone predominantly secreted by EECs, GLP-1 not only governs systemic energy homeostasis but also engages in bidirectional communication with the ENS via synapse-like contacts and paracrine signaling, thereby exerting integrative control within the epithelial–neural microenvironment [55].

Beyond incretin hormones, PYY—primarily secreted by EECs, particularly L cells—acts as a critical inhibitory neuropeptide within the ENS–EEC network. By binding to Y2 receptors on enteric neurons, PYY suppresses neuronal excitability, thereby modulating intestinal motility and immune responses [56]. This inhibitory function positions PYY as a key “braking signal” essential for maintaining intestinal homeostasis. In CRC, PYY expression is frequently diminished, and reduced PYY levels have been linked to tumor progression and unfavorable prognosis [57]. Functional studies further indicate that PYY suppresses CRC cell proliferation, migration, and invasion while promoting apoptosis. Moreover, exogenous PYY administration inhibits tumor organoid growth, underscoring its potential as a therapeutic target [58].

The gut microbiota, acting as a dynamic and plastic “exogenous regulatory layer,” continuously reshapes the EEC–ENS axis through metabolite-driven signaling and integrates into the CRC-associated neuro–immune–metabolic network [59]. At the compositional level, commensal and pathogenic bacteria exert opposing functional effects. For example, Akkermansia muciniphila, a representative beneficial symbiont, maintains mucosal barrier integrity and produces metabolites such as acetate and propionate, which enhance EEC-derived GLP-1 secretion, improve epithelial barrier function, and modulate ENS neuronal excitability—collectively reflecting a potentiating effect on the EEC–ENS axis [60]. In contrast, pro-inflammatory species such as Fusobacterium nucleatum promote chronic inflammation via activation of the TLR4/NF-κB pathway and reprogram the local metabolic milieu, thereby suppressing physiological gut hormone secretion and fostering an immunosuppressive microenvironment. These effects disrupt the homeostatic coupling of the EEC–ENS axis and drive CRC progression, including microsatellite instability–associated phenotypes [61]. At the metabolite level, short-chain fatty acids (SCFAs) serve as central mediators linking the microbiota to the host neuroendocrine system. SCFAs activate FFAR2/3 receptors on EECs, stimulating the secretion of GLP-1 and PYY while regulating EEC differentiation and ENS excitability [62]. Additionally, tryptophan-derived microbial metabolites (e.g., indole derivatives) enhance EEC function via activation of the aryl hydrocarbon receptor (AhR) and may further influence lineage specification through modulation of the Wnt/Notch axis, thereby amplifying their regulatory impact on both immune and neural systems [63].

**Fig. 2:**
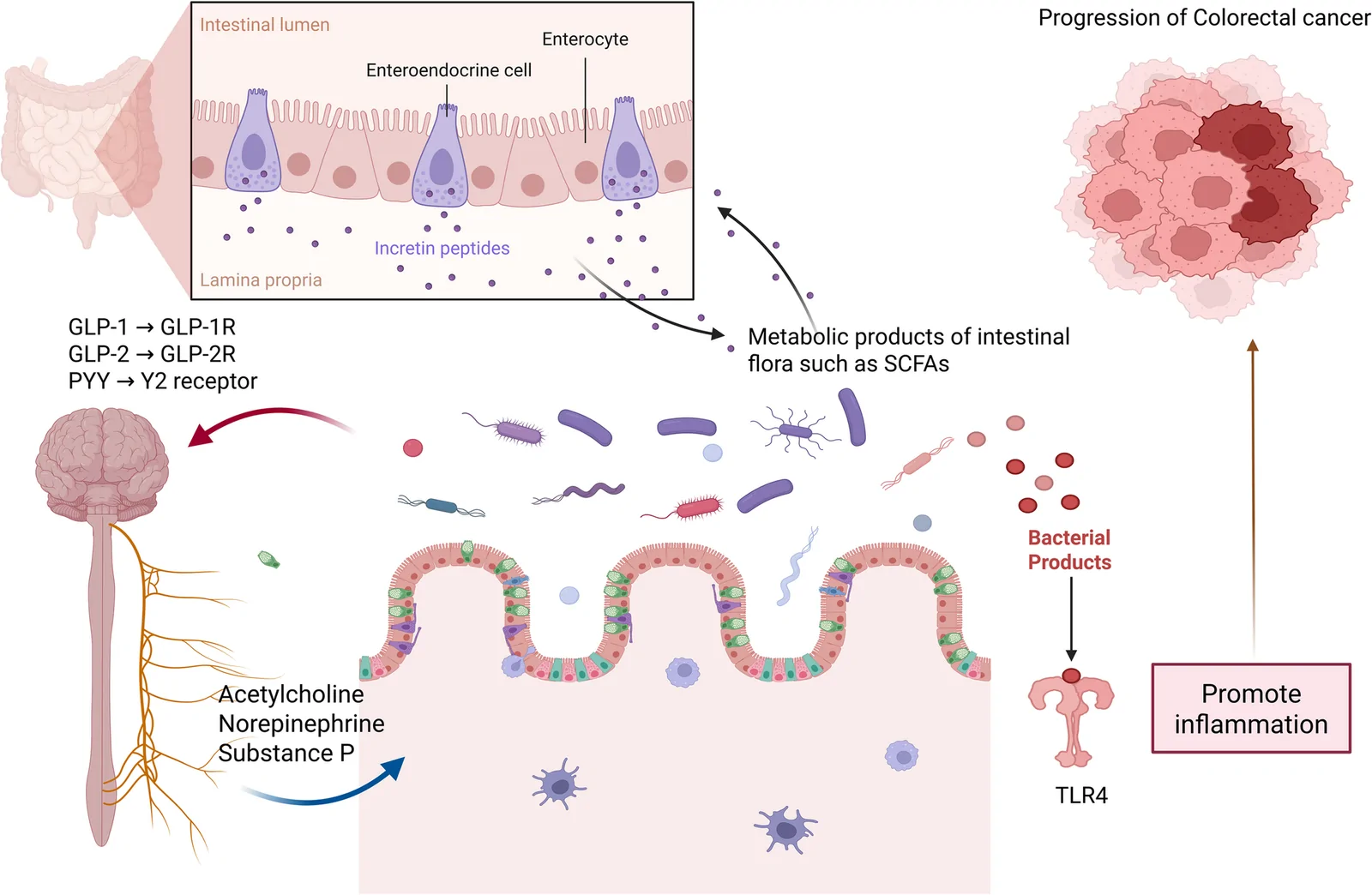
Role of enteroendocrine cells and neurotransmitters in colorectal cancer progression. Enteroendocrine cells (EECs) contribute to colorectal cancer progression through the secretion of incretin peptides (GLP-1, GLP-2, PYY) and by mediating responses to gut microbiota-derived metabolites, such as short-chain fatty acids (SCFAs). Bacterial products signal through Toll-like receptor 4 (TLR4), further amplifying intestinal inflammation. Meanwhile, neurotransmitters including acetylcholine, norepinephrine, and substance P act on intestinal receptors to modulate mucosal immune responses and promote inflammatory processes, thereby facilitating colorectal cancer progression

Collectively, the EEC–ENS axis does not function as an isolated regulatory module but rather constitutes a highly dynamic neuro–endocrine–immune network shaped by serotonergic signaling, tumor cell plasticity, and microbiota-derived metabolic inputs (Fig. 2). This integrated system not only drives intrinsic tumor cell reprogramming but also orchestrates tumor microenvironment remodeling, thereby influencing CRC initiation and progression. These insights highlight the EEC–ENS axis as a promising systems-level target for future therapeutic intervention in CRC.

#### The central nervous system's cross-organ regulatory axis—the quadruple dialogue of brain-gut-microbiota-tumor network

The CNS regulates CRC through a multidimensional signaling network, with the brain-gut axis (GBA) as the core hub. This axis integrates the CNS, the autonomic nervous system (ANS), the ENS, and the gut microbiota to form a dynamic interaction. Ultimately, it constitutes a quadruple regulatory network of brain-gut-microbiota-tumor through "stress signaling-metabolite conduction-receptor-mediated effects," profoundly influencing the occurrence and progression of CRC. CRC patients often experience chronic stress, which, over time, activates the sympathetic nervous system and the hypothalamic–pituitary–adrenal (HPA) axis, leading to increased release of catecholamines and glucocorticoids [64]. Chronic stress can enhance CRC cell glycolysis through the β₂-AR/CREB1 signaling pathway, promoting tumor progression: for example, stress-induced NE can activate the IL-6/STAT3 pathway through the β₂-AR/PKA/CREB1 axis, exacerbating immune suppression in TME [65]. CRC disturbs the HPA axis through pro-inflammatory cytokines (such as IL-6) and vagal afferent signals, leading to elevated systemic glucocorticoid levels, thereby systemically suppressing anti-tumor immunity. In chronic stress mouse models, stress-induced Epi can promote CRC EMT and maintain CRC cell stemness through the CEBPB/TRIM2/P53 axis, while also enhancing p53 protein ubiquitination and degradation, reducing its stability [66]. In contrast, acute stress appears to be more beneficial in reducing tumor burden.

Emerging evidence highlights the ability of gut microbiota to modulate anti-tumor immune responses, either directly or indirectly, via their metabolic byproducts. Among these metabolites, short-chain fatty acids (SCFAs), particularly butyrate, along with amino acid derivatives such as tryptophan and glutamate metabolites generated from microbial fermentation of dietary fiber, function not only as local signaling molecules that shape the immune microenvironment but also as systemic regulators acting through the gut-brain axis [67]. Butyrate, in particular, is critically involved in preserving intestinal barrier integrity, modulating inflammatory responses, and supporting metabolic homeostasis. Acting through GPR43/109A receptor-dependent pathways, butyrate exerts dual regulatory effects. Centrally, it stimulates vagal afferent fibers in the gut, suppresses excessive HPA axis activity, and reduces the release of stress hormones like epinephrine, thereby indirectly restraining CRC progression [68]. Locally, within the colonic tumor microenvironment, butyrate promotes regulatory T cell differentiation while dampening Th17 cell polarization, thus alleviating IL-17-driven chronic inflammation. Additionally, it limits dendritic cell overactivation, preventing IL-27-mediated CD8⁺ T cell exhaustion and thereby sustaining anti-tumor immune homeostasis [69].

**Fig. 3:**
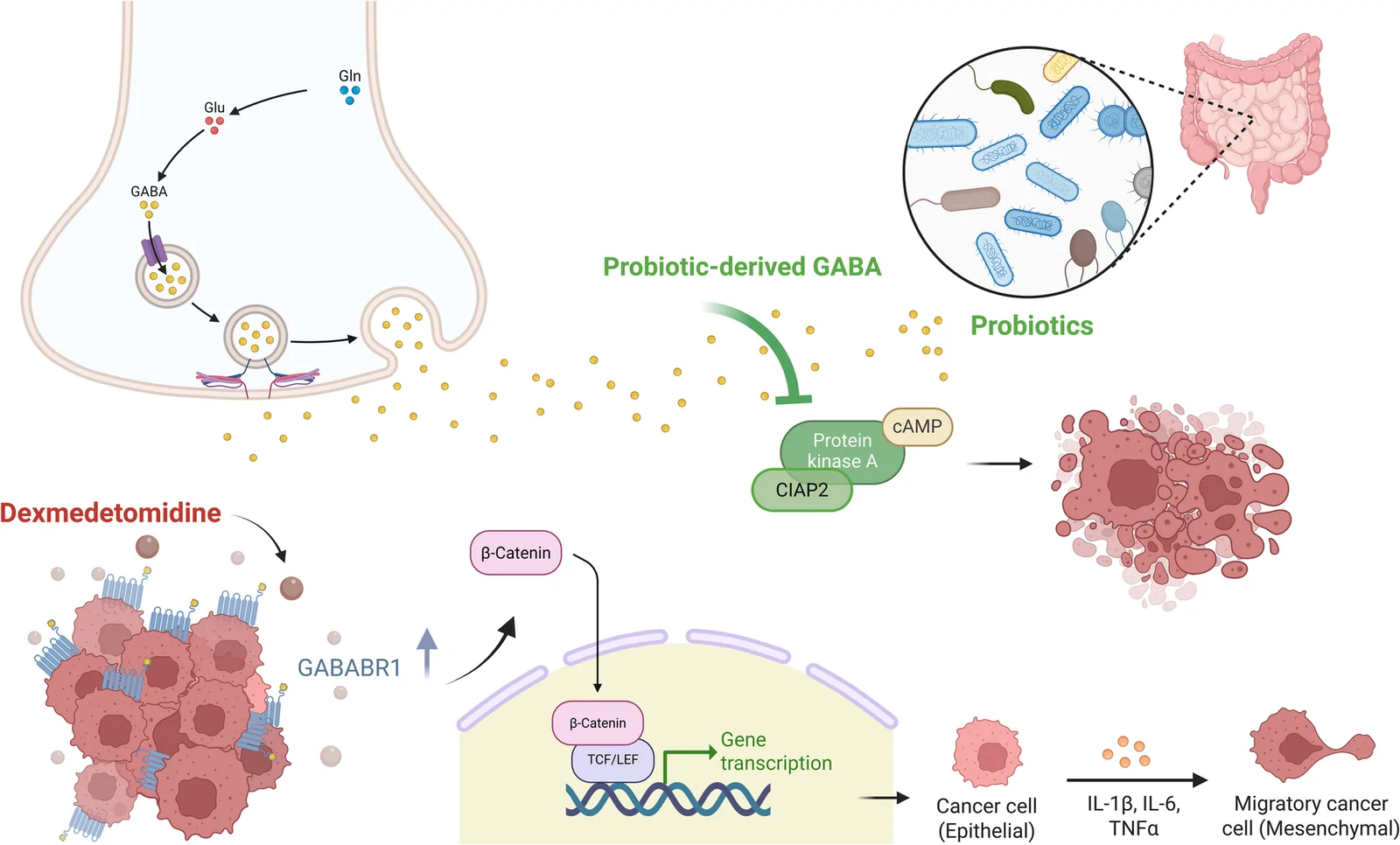
GABA signaling in colorectal cancer: a double-edged sword. Probiotic-derived GABA activates GABABR, inhibits cAMP/PKA, and downregulates cIAP2 to induce apoptosis. Conversely, dexmedetomidine (DEX) upregulates GABABR1, activates β-catenin signaling, and promotes EMT-driven cancer cell migration

Gamma-aminobutyric acid (GABA), the principal inhibitory neurotransmitter in the central nervous system, is synthesized via a glutamine metabolic pathway catalyzed by glutaminase (GLS). In this process, glutamine is converted to glutamate by GLS, which is subsequently decarboxylated to form GABA by glutamate decarboxylase (GAD). Recent studies have shown that GABAergic signaling undergoes significant reprogramming in CRC, with abnormal accumulation of GABA closely linked to tumor progression (Fig. 3). This signaling also exhibits a receptor subtype-dependent "double-edged sword" effect [70]. This functional differentiation suggests that GABA does not solely regulate tumor behavior in a unidirectional manner, but instead mediates distinct biological outcomes depending on the specific receptor context. In terms of promoting tumor progression, GABA can enhance CRC invasiveness through particular signaling axes. For example, the α₂-adrenergic receptor agonist dexmedetomidine (DEX) has been shown to dose-dependently elevate GABA release in CRC cells and upregulate the GABAB receptor 1 subunit (GABABR1, GB1e). This, in turn, activates β-catenin signaling and promotes EMT, thereby markedly enhancing tumor cell proliferation, migration, and invasive capacity [71]. In contrast, GABAB receptors (GABABR) are generally expressed at low levels in CRC, and their loss or functional inhibition is closely associated with a more malignant tumor phenotype. Mechanistically, GABABR knockout induces upregulation of EMT markers, such as N-cadherin and vimentin, along with the loss of E-cadherin. GABABR1, on the other hand, can exert tumor-suppressive effects by inhibiting EMT and the Hippo/YAP1 signaling pathway, thus restricting CRC progression and metastasis [66]. Therefore, the functional role of GABAergic signaling depends on the fine regulation of receptor subtypes and their downstream pathways. In light of this, targeted interventions in the GABAergic axis have garnered increasing attention. For example, GABA-producing probiotics, such as Lactobacillus species, can activate the GABAB receptors on tumor cells, downregulating the cAMP/PKA signaling pathway and exerting antitumor effects. This approach induces tumor cell apoptosis and, simultaneously, reverses 5-fluorouracil (5-FU) resistance by decreasing the expression of the anti-apoptotic protein CIAP2 [72]. These findings suggest that a "microbe-metabolite-receptor" axis-based regulatory strategy may provide new insights and intervention targets for the comprehensive treatment of CRC [73].

In summary, CRC is regulated by the nervous system, involving multiple neural subsystems, including the sympathetic, parasympathetic, sensory, and ENS (Table 2). Neurotransmitters such as epinephrine (Epi), along with neurotrophic factors including NGF, exert a significant influence on tumor progression. These molecules activate specific receptors and downstream signaling cascades, thereby promoting malignant cell proliferation, migration, and invasion. Beyond these direct effects, the nervous system also engages neuroendocrine pathways and immune system crosstalk to exert regulatory functions. The gut–microbiome–brain axis, for example, contributes to CRC modulation through integrated neuroendocrine–immune circuits. Collectively, these diverse neural influences on colorectal tumor biology highlight the nervous system as a promising therapeutic target in oncology.

**Table 2:** Neural subsystem regulation mechanisms and potential tumor outcomes

Neural Subsystem	Key Molecules/Signals	Immunological Effects	Tumor Outcomes (Proposed Tumor Outcomes)
Parasympathetic Nerve	Acetylcholine (ACh)	Decreased TNF-α, IL-1β, IL-6, IL-18	Inhibition of inflammation-driven tumorigenesis
Parasympathetic Nerve	Vagotomy	Reduced cholinergic inhibition	Confirmation of the protective role of cholinergic signaling
Sympathetic Nerve	Norepinephrine (NE)	Increased PD-1, MDSC, T cell exhaustion	Promotion of immune evasion and tumor progression
Sympathetic Nerve	NE-β2-AR blockade	Enhanced cytotoxicity, mitochondrial activity	Enhanced response to immunotherapy
Sensory Nerve	Substance P (SP)	Enhanced NK cell function, increased active T cell survival	Enhanced anti-tumor immunity
Sensory Nerve	CGRP	Decreased pro-inflammatory factors, increased IL-10	Suppression of anti-tumor immunity
Sensory Nerve	GABA	Decreased IL-6, reduced cytotoxicity	Promotion of tumor immune evasion
Central Nervous System	GABA	Reduced anti-tumor immunity	Accelerated tumor growth in colon cancer models
Central Nervous System	Schwann cells (SC)	Recruitment of MDSC, increased immunosuppression	Promotion of melanoma progression

### CRC-induced remodeling of the nervous system

#### Neural invasion and tumor microenvironment metabolic reprogramming

Perineural invasion (PNI) is a biological hallmark of the aggressive invasiveness of CRC, essentially representing the ability of CRC cells to overexpress key neurotrophic factors and construct a "signaling network" that promotes their own invasion and neural remodeling. Clinical studies have confirmed that PNI is directly associated with increased depth of CRC invasion, elevated risk of metastasis, and poor prognosis, serving as a key distinguishing indicator for the highly invasive subtypes of CRC [74]. The overexpression of NGF and BDNF represents a key initiating event in this process. By binding specifically to TrkA and TrkB receptors on neuronal surfaces, these neurotrophic factors not only activate intracellular proliferative signaling pathways in CRC cells and suppress apoptosis-related proteins, thereby enhancing tumor cell survival [75], but also promote aberrant synaptic sprouting and neural remodeling. This leads to the formation of structurally disorganized “neural tracks” that provide anatomical conduits for cancer cell dissemination along the perineural space [76]. More critically, the recruited neural elements release various neurotransmitters through retrograde signaling, further accelerating tumor progression. For instance, the presence of NE within TME promotes pseudopodia formation and enhances motility in CRC cells via the β-AR/cAMP/PKA axis. Additionally, NE upregulates Foxp3 expression in T cells by activating the cAMP response element-binding protein (CREB) pathway, which facilitates regulatory T cell differentiation, suppresses CD8⁺ T cell activity, and increases the production of TGF-β and IL-10, collectively fostering an immunosuppressive microenvironment [77]. The complex crosstalk among cellular and molecular constituents of TME plays a pivotal role in driving PNI and facilitating CRC advancement. While traditional views emphasized tumor cells, immune cells, cancer-associated fibroblasts, vascular components, and the extracellular matrix as core elements of the TME, emerging evidence indicates that neural components now represent an essential functional part of this microenvironment [78]. NE performs functions beyond those already discussed. By acting on α2-AR located on the macrophage surface, it blocks M1 polarization and facilitates the shift toward the M2 phenotype. One consequence of this change is a decrease in the secretion of anti-tumor factors, including IL-12 and TNF-α. In parallel, nerve terminals release neuropeptides such as substance P. These molecules activate pain signaling pathways, which in turn produce cancer-related pain in CRC patients [79]. In summary, neuroimmune interactions form a multi-level regulatory network within the TME, not only promoting the occurrence of PNI but also providing a potential mechanism for CRC immune evasion.

Increased sympathetic nerve activity in the TME leads to the release of NE, which induces vasoconstriction and triggers local hypoxia, thereby activating hypoxia-inducible factor HIF-1α. HIF-1α further upregulates glycolysis-related genes (such as LDHA and GLUT1), shifting glucose metabolism towards glycolysis and resulting in the accumulation of large amounts of lactic acid. Lactic acid, beyond being a glycolytic byproduct, actively modulates histone lactylation within the promoter regions of pivotal glycolytic enzymes (e.g., HK-1) and TCA cycle-associated genes (e.g., IDH3G), thereby altering transcriptional activity and establishing a self-reinforcing feedback mechanism that perpetuates glycolytic reprogramming [80]. Under conditions of sustained hypoxia, glucose scarcity, and lactate accumulation, tumor cells activate lipid metabolic remodeling—comprising increased lipid uptake, synthesis, and oxidation—to sustain energy production and redox balance. Concurrently, lipid buildup markedly impairs immune cell functionality within the TME: linoleic acid, for instance, promotes mitochondrial oxidative stress and cell death in CD4⁺ T cells via upregulation of CPT1A; meanwhile, elevated lipid levels in TAMs can trigger CD36 overexpression in CD8⁺ T cells, resulting in lipid peroxidation and functional impairment [81]. This coordinated metabolic reprogramming mechanism not only supports the malignant progression of CRC but also provides a new perspective for therapeutic strategies targeting the neuro-metabolic-immune axis.

#### The brain-gut axis in upstream regulation and paraneoplastic syndromes

The brain-gut axis constitutes a sophisticated bidirectional communication network that links the gastrointestinal tract with the CNS, coordinating inputs from the nervous, endocrine, and immune systems. This system is critically implicated in the initiation, progression, and systemic manifestations of CRC. Its functionality depends on the integrated actions of the ANS, the ENS, and the intestinal microbiota. ANS dysregulation may disrupt brain-gut homeostasis, often concurrent with microbial imbalance, thereby influencing CRC pathogenesis. Throughout CRC development, tumor-induced mechanical compression and localized inflammatory mediators—including IL-1β and TNF-α—stimulate vagal afferent terminals. These signals are relayed through the nucleus tractus solitarii (NTS) to hypothalamic and amygdalar regions, modulating both appetite and affective states [82]. By crossing the blood–brain barrier, circulating pro-inflammatory cytokines including IL-6 and TNF-α reach the hypothalamic paraventricular nucleus (PVN). There, they trigger the activation of the hypothalamic–pituitary–adrenal (HPA) axis, leading to the sustained release of glucocorticoids. This state does not merely precipitate systemic metabolic dysregulation and depressive-like behaviors; it also promotes CRC metastasis. The pro-metastatic effect is mediated by synergistic crosstalk between glucocorticoid receptors and epigenetic regulators such as TET2 [83]. In addition, CRC can lead to increased levels of ghrelin and suppressed leptin signaling, disrupting energy homeostasis and exacerbating cancer-related anorexia and cachexia [84].

CRC can also indirectly affect the nervous system through immune cross-reactivity, known as paraneoplastic neurological syndromes (PNS). PNS is a group of neurological diseases caused by abnormal immune reactions induced by tumors, which are rare manifestations of cancer. From the perspective of antigen targeting, the key pathological basis of PNS is the molecular mimicry between specific antigens expressed by CRC cells and neuronal surface antigens, which in turn activates the immune system to produce autoantibodies targeting neuronal antigens. Antibodies targeting intracellular antigens mainly mark T cell-mediated neuronal damage, while antibodies targeting cell surface antigens can directly cause neuronal dysfunction through receptor blockade or internalization [85]. The clinical manifestations of PNS are highly complex and diverse, covering a wide range of conditions, including CNS disorders (such as limbic encephalitis), peripheral nervous system disorders (such as autonomic neuropathy), and neuromuscular junction diseases (such as Lambert-Eaton myasthenic syndrome). The specificity of symptoms is closely related to the spectrum of autoantibodies, with different antibody subtypes often corresponding to characteristic patterns of neurological dysfunction. In addition to the direct action of autoantibodies, cytokines from TME also participate in the occurrence of neurological symptoms. For example, GDF15, which is significantly elevated in the serum and tissues of CRC patients, can trigger anorexia-cachexia syndrome by binding to the GFRAL receptor in the area postrema of the brainstem, and its levels are closely related to poor prognosis [86]. Of note, PNS-related neurological symptoms—for instance, unexplained progressive cerebellar ataxia, limb numbness and weakness, and cognitive impairment—may precede gastrointestinal tumor manifestations in certain CRC cases. Consequently, these neurological signs could serve as early warnings for CRC, offering key clinical clues that facilitate the identification of otherwise occult colorectal malignancies [87].

More broadly, CRC can trigger systemic immune-neural interactions that are amplified along the "gut-immune-brain axis," ultimately leading to central neuroinflammation and functional remodeling. The cachectic state associated with CRC can cause changes such as microglial/macrophage activation, astrocyte functional spectrum disorder, and mTOR pathway-related neuroinflammation [88]. In inflammatory bowel disease models, gut immune cells migrate to the cerebrospinal fluid via the choroid plexus or meningeal endothelium, activating the TLR4/NF-κB pathway in microglia and promoting the release of IL-1β and TNF-α, leading to depression-like behaviors [89]. Persistent inflammation weakens the ability of astrocytes to take up glutamate, resulting in the accumulation of glutamate in the synaptic cleft. This accumulation, mediated by NMDA receptors, causes prefrontal neuronal damage, which is closely related to anxiety and cognitive impairment [90]. In addition, recent studies have shown that survivors of CRC often experience maladaptive neuroplasticity changes in the medial temporal lobe, which are significantly correlated with declines in verbal memory function [91].

## Targeted intervention strategies based on the bidirectional communication between CRC and the nervous system

### Denervation intervention: severing the tumor-neural "communication" ties

Peripheral nerve infiltration is critical for the advancement and metastatic spread of CRC. Denervation strategies seek to interrupt detrimental communication between neural components and malignant cells, representing an emerging treatment modality. Both surgical and chemical denervation have demonstrated potential in preclinical and selected clinical settings. Surgical methods allow precise excision of tumor-innervating nerve fibers, thereby diminishing the provision of neurotrophic growth factors and suppressing oncogenic signaling within TME. For example, in gastric cancer investigations, bilateral vagotomy markedly decreased the incidence and expansion of gastric tumors in murine models, whereas unilateral vagotomy solely restrained ipsilateral tumor development. Vagus nerve stimulation (VNS), a non-destructive neuromodulatory approach, also exhibits possible synergistic antitumor benefits [92]. Although evidence in CRC remains scarce, studies in glioblastoma models indicate that electrical vagus nerve stimulation can promote T-cell immune activation via the cholinergic α7 nicotinic ACh receptor (α7nAchR), systemically repress IL-6, along with other inflammatory mediators such as IL-1β and TNF-α, thereby converting immunoresistant malignancies into immunoresponsive ones and arresting glioma progression [93]. Chemical denervation involves locally applying neurotoxic drugs to inhibit neurotransmitter release from nerve terminals or disrupt nerve fiber structure, thereby blocking tumor neural signaling. Pharmacological inhibition and genetic knockout of the muscarinic ACh M3 receptor have shown inhibitory effects on gastric, small intestinal, and breast tumors. Thus, botulinum toxin (BoNT), which blocks ACh release, holds broad application prospects, as it has been proven to inhibit tumor growth [94]. Moreover, BoNT/A, by inhibiting ACh release from nerve terminals, cuts off the stimulatory signals from the vagus nerve to the tumor and effectively relieves muscle spasms caused by cancerous masses, reducing postoperative neuropathic pain at surgical and radiation sites [95].

### Interfering with intratumoral neural signaling

#### Targeting the serotonin axis to reshape anti-tumor immunity

Dual roles are played by serotonin (5-HT) within the CRC tumor microenvironment, where it both regulates immune responses and affects tumor cell biology. Following tumor antigen recognition, 5-HT is secreted by CD8⁺ T cells, which in turn activates the MAPK-TCR pathway, leading to enhanced anti-tumor activity. This activation process is negatively regulated by the serotonin transporter (SERT), which reuptakes extracellular 5-HT into cells, thereby reducing its concentration in the TME. When SERT is blocked by selective serotonin reuptake inhibitors (SSRIs) such as fluoxetine, intratumoral 5-HT accumulation occurs. This accumulation results in significantly boosted CD8⁺ T cell activity and enhanced anti-tumor immunity, positioning SSRIs as promising candidates for CRC immunotherapy [96]. High expression of the 5-HT receptors HTR1B, HTR1D, and HTR1F is observed on CRC stem cells. Upon 5-HT binding, the Wnt/β-catenin signaling pathway is activated by these receptors, which promotes cancer stem cell proliferation and tumorigenesis. Effective suppression of CRC stem cell proliferation and tumor growth can be achieved by knocking out these receptors or by blocking them with antagonists [97].

#### Inhibiting adrenergic signaling to curb tumor progression

CRC cells exhibit substantial surface expression of ADRB2. Activation of the β-catenin/ZEB1 signaling axis—a central pathway implicated in tumor proliferation, migration, and metastasis—occurs following binding by NE, which is released from sympathetic nerve terminals. Significant anti-tumor activity has been demonstrated through pharmacological inhibition of ADRB2, with propranolol—a non-selective β-blocker—being the most extensively studied agent. The effects of propranolol depend on cellular context, drug concentration, and culture conditions; it can induce cell death, alter cell cycle progression, and modulate intracellular and mitochondrial reactive oxygen species (ROS) levels. At higher concentrations, propranolol promotes ROS-mediated apoptosis [24]. Notably, the sympathetic nervous system undergoes dynamic degeneration during CRC progression. Using a DSS/AOM-induced CRC mouse model, Chen and colleagues observed that the density of sympathetic nerve fibers surrounding tumors gradually declines as the disease advances, and this degenerative process correlates closely with worsening of the immune microenvironment [70]. More specifically, sympathetic neurodegeneration reduces norepinephrine secretion and leads to insufficient activation of α₂-adrenergic receptors (ADRA2). This results in a 32% decrease in CD8⁺ T cell recruitment, exacerbates the immunosuppressive microenvironment, and ultimately accelerates CRC malignancy [98]. These findings point to ADRA2 as a potential target for CRC immune modulation, complementing ADRB2 as part of a dual-pronged approach to adrenergic signaling intervention. On one side, ADRB2 blockade inhibits the malignant phenotype of tumor cells; on the other, moderate activation of ADRA2—using selective ADRA2 agonists, for example—enhances CD8⁺ T cell recruitment and improves the immune microenvironment.

#### Targeting neuropeptides to block pro-tumorigenic signals

In CRC progression, neuropeptides act as key signaling molecules within the tumor–neuro–immune network. By regulating critical processes such as angiogenesis, immune microenvironment remodeling, and EMT, neuropeptides become significant drivers of tumor progression and treatment resistance [99]. These small peptides function in an autocrine or paracrine manner to influence tumor cells, immune cells, and stromal cells, forming a "pro-cancer signaling loop." They not only directly promote tumor proliferation and invasion but also facilitate immune escape by fostering an immunosuppressive milieu. Accordingly, therapeutic strategies targeting neuropeptides and their cognate receptors have emerged as a promising avenue for CRC-directed intervention (Table 3).

**Table 3:** Neuroregulatory targets and mechanisms of related drugs

Target	Representative Drug	Mechanism of Action
β-Adrenergic Receptor (β-AR)	Propranolol	Non-selective β-blocker, inhibits NE-mediated cAMP/PKA pathway, reverses immunosuppressive microenvironment (reduces Treg, MDSC)
Muscarinic Acetylcholine Receptor (mAChR)	Botox (Botulinum Toxin Type A)	Inhibits cholinergic nerve terminals from releasing ACh, blocks ACh transactivation of EGFR/ERK oncogenic pathways via mAChR (e.g., M3R)
Neurotrophic Factor Receptor (Trk)	Larotrectinib	Highly selective TrkA/B/C inhibitor, blocks NGF/BDNF-mediated MAPK/ERK and PI3K/AKT pathways, inhibits tumor proliferation and innervation
CGRP Receptor	CGRP Receptor Antagonist (e.g., Olcegepant)	Blocks CGRP signaling released by sensory neurons, inhibits cAMP-PKA pathway-driven upregulation of VEGF/MMP-9
AMPA Glutamate Receptor	Perampanel	Non-competitive AMPA receptor antagonist, inhibits glutamate-mediated Ca^2^⁺ influx and PI3K/AKT activation
DRD2 Dopamine Receptor	ONC201	Antagonizes DRD2/3 dopamine receptors, inhibits Akt-ERK pathway and targets cancer stem cells
Neuroimmune Cross-target	Anti-NGF Antibody (Tanezumab)	Neutralizes NGF to block TrkA activation, reduces perineural invasion and reverses immunosuppression

Substance P (SP), primarily secreted by sensory neurons and tumor cells, binds to its high-affinity receptor, neurokinin 1 receptor (NK1R), activating the PKC/NF-κB signaling pathway and significantly upregulating vascular endothelial growth factor (VEGF) expression, thereby promoting tumor angiogenesis. In terms of immune regulation, SP induces M2 polarization of macrophages and inhibits the infiltration and function of cytotoxic T cells, creating an immunosuppressive microenvironment conducive to tumor growth [100]. Notably, aprepitant—an NK1R antagonist already approved as an antiemetic—shows anti-tumor activity in preclinical CRC models. It inhibits the SP/NK1R signaling pathway, which reduces microvascular density and TAM recruitment. These changes subsequently suppress tumor growth. In a separate approach, combining thalidomide with Clostridium butyricum boosts the anti-cancer effect of cisplatin. This combination works by activating the caspase-3-mediated apoptotic pathway. At the same time, it reduces chemotherapy-induced nausea and vomiting and also slows tumor progression in both the brain and colon. All these benefits come from suppressing the tachykinin 1/NK-1R signaling axis. Thus, this strategy offers new possibilities for combination therapy in CRC [101].

**Fig. 4:**
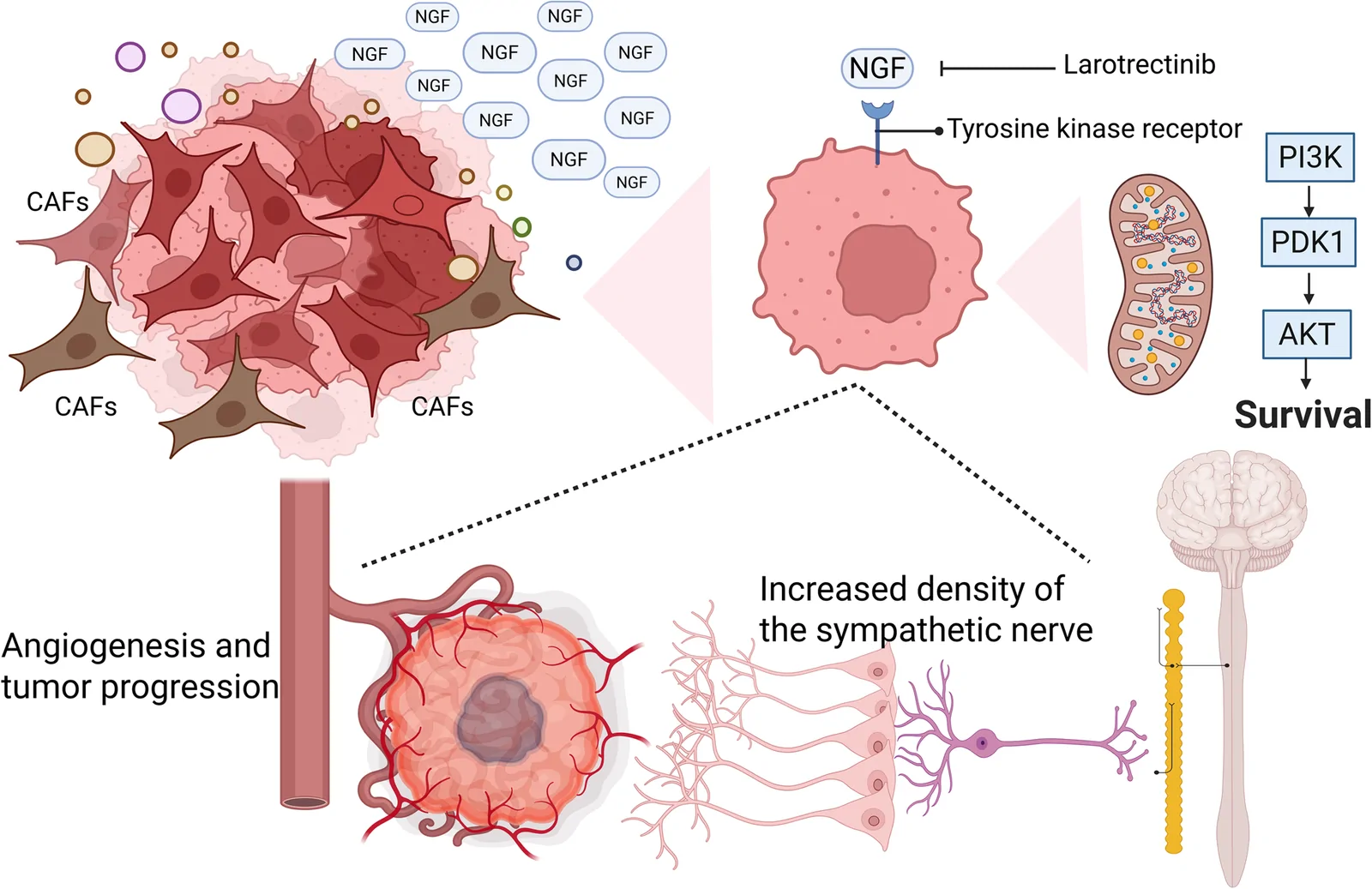
Cancer-associated fibroblasts promote a vicious cycle of sympathetic nerve-related cancer cell malignancy. Under the stimulation of norepinephrine (NE), cancer-associated fibroblasts (CAFs) can secrete large amounts of nerve growth factor (NGF). After NGF binds to its receptor, it not only promotes the regeneration of sympathetic nerve fibers within the tumor but also exacerbates angiogenesis and tumor progression. The newly generated sympathetic nerves, in turn, release more NE under stress, forming a vicious cycle that promotes tumor development

Under the influence of NE, cancer-associated fibroblasts (CAFs) are stimulated to secrete substantial quantities of NGF (Fig. 4). Following binding to tyrosine kinase receptors, NGF facilitates both the regeneration of sympathetic nerve fibers inside the tumor and triggers mitochondrial metabolic reprogramming via activation of the PI3K–AKT signaling pathway. These processes collectively enhance angiogenesis and accelerate tumor progression [102]. Preclinical investigations have demonstrated that the broad-spectrum TRK inhibitor larotrectinib can concurrently suppress neural innervation and angiogenic activity, leading to marked inhibition of CRC growth and metastasis. This evidence supports the targeting of neuro-angiogenic crosstalk as a promising combinatorial therapeutic approach [103]. In CRC cells with SMARCA1 deficiency, NPFF secretion is significantly increased, which activates the JAK2–STAT5 signaling pathway in an autocrine/paracrine manner, inducing EMT and enhancing the invasive capacity of tumor cells. Moreover, NPFF has strong immunomodulatory functions: it promotes M2 polarization of macrophages and upregulates various pro-tumorigenic cytokines, thereby shaping an immunosuppressive microenvironment [104]. This mechanism suggests that targeting the SMARCA1–NPFF axis could not only inhibit metastasis but also reverse immune resistance, offering new treatment options for patients unresponsive to immunotherapy [105].

### Other potential targeted directions

#### Targeting Neuregulin-1 (NLG1)

Neuregulin-1 (NLG1), a type I transmembrane protein, binds autocrinely to its synaptic ligand neurexin in CRC. This binding facilitates the membrane localization of antigen-presenting cells, activates the β-catenin/WNT signaling pathway, and upregulates both mesenchymal markers and WNT-responsive genes, thereby augmenting the migratory and invasive properties of tumor cells [106]. Elevated NLG1 expression has been strongly associated with increased lymph node metastasis and unfavorable clinical outcomes in CRC. Treatment with NLG1-targeting monoclonal antibodies results in the downregulation of mesenchymal markers (e.g., vimentin), suppression of cell migration, and inhibition of the exosome-mediated anti-inflammatory immune response within the TME, positioning NLG1 as a promising surface-exposed therapeutic target in CRC [107].

#### The dual role of Schwann Cells (SCs) and targeting

**Fig. 5:**
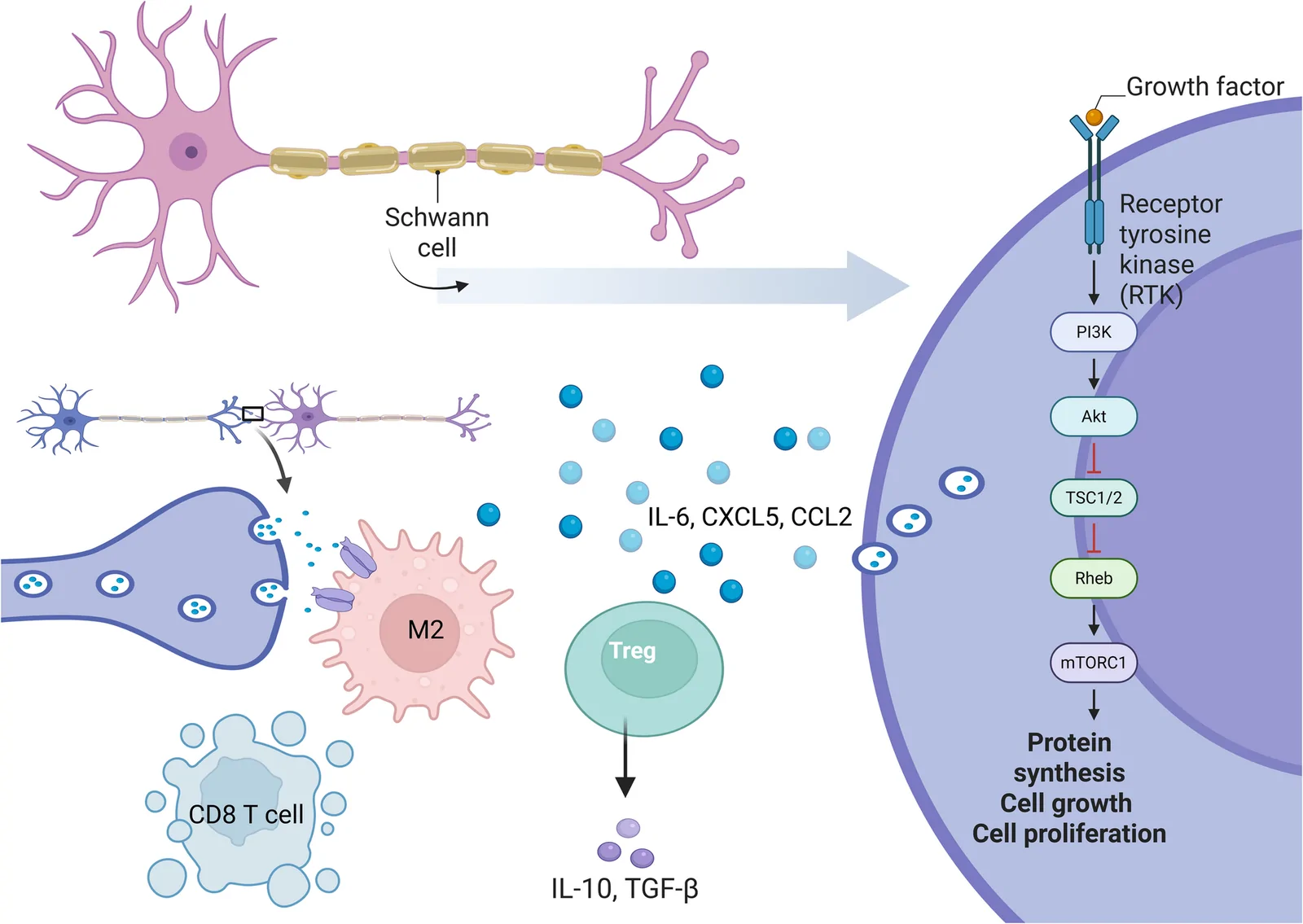
The interaction network between Schwann cells and CRC cells. Schwann cells (SCs) activate the two key oncogenic signaling pathways, RAS-MAPK and PI3K-AKT, within cells through the secretion of factors such as CCL2 and IL-6. Meanwhile, these signals reprogram the tumor microenvironment in a STAT3-dependent manner. These changes drive tumor-associated macrophages (TAMs) to polarize towards the M2-like pro-tumorigenic phenotype and induce the expansion of myeloid-derived suppressor cells (MDSCs), leading to CD8⁺ T cell exhaustion and ultimately creating a vicious cycle that synergistically promotes tumorigenesis and immune evasion

Schwann cells (SCs), as peripheral glial cells, are highly plastic and multifunctional, making them easily hijacked and utilized by cancer cells (Fig. 5). On one hand, tumor-induced repair-like SCs can reshape the extracellular matrix, secrete cytokines to recruit and reprogram immune cells, and accelerate tumor growth. On the other hand, SCs may also play immunomodulatory roles that inhibit tumor growth under specific conditions [108]. Tumor-associated Schwann cells (TAScs) have emerged as key players in the progression of highly innervated cancers and are associated with perineural invasion, making them potential new therapeutic targets in non-neural cancers [109]. Additionally, denervation-associated Wallerian-like degeneration at the tumor invasive front, accompanied by functional alterations in Schwann cells (SCs), may limit tumor nerve infiltration and indirectly remodel the tumor microenvironment through effects on immune cells, fibroblasts, and angiogenesis. Furthermore, the involvement of SCs in cancer-associated pain and potential paraneoplastic syndromes remains an important area requiring further investigation [110]. Therefore, SCs hold promise as new intervention targets in CRC treatment.

#### Blocking exosome-mediated neuro-tumor interactions

Exosomes are nanoscale lipid vesicles universally secreted by cells, and their cargo faithfully reflects the molecular characteristics of the parent cells [111]. Neuronal exosomes are rich in axon guidance molecules and can remotely construct a "pre-metastatic niche" for neuro-tumor interactions, promoting tumor innervation. In contrast, exosomes secreted by tumor cells can actively recruit axons and accelerate nerve fiber growth within the tumor [112]. Given the decisive role of cancer-derived exosomes in axonogenesis, strategies targeting their biogenesis, release, or functional blockade may represent a new generation of precision therapies for intervening in neuro-tumor co-evolution. For example, LINC00183 is an exosome (PLT-Exos) derived from platelets in CRC patients. It promotes the malignant biological behavior of CRC cells in vitro by binding to and stabilizing enolase 1 (ENO1), thereby enhancing glycolysis and leading to H3K18 lactylation and transcriptional activation of the oncogene GDF15 [113]. Strategies targeting exosome biogenesis, release, and functional blockade, such as using the exosome biogenesis inhibitor GW4869, can significantly reduce neuronal exosome release, which may be helpful or crucial for tumor progression [114]. However, the routes of administration and safety of these inhibitors still need further evaluation, and the effects of other exosome inhibitors in neuro-tumor interactions need to be compared [115].

### Combination with immunotherapy

The core reason for immunotherapy resistance in CRC is the "immunosuppressive state" of the TME, which includes insufficient infiltration of effector T cells, enrichment of regulatory T cells and M2-type macrophages, and high expression of immune checkpoint molecules (such as PD-L1). Propranolol, by blocking ADRB2 signaling, can reshape the immune-activated state of the TME from two aspects: promoting effector T cell function and inhibiting immunosuppressive cells, thereby improving the response to immunotherapy. Within the bidirectional regulatory network connecting CRC to the nervous system, neuro-immune imbalance has emerged as a key therapeutic target. Restoring this balance could help reverse the immunosuppressive state of the TME and enhance responses to immunotherapy. Strategies aimed at this regulatory axis rely chiefly on precise modulation of sympathetic and parasympathetic neural pathways. Specifically, β-adrenergic receptor antagonists (e.g., propranolol) inhibit excessive sympathetic activation within TME. Through suppression of β2 receptor-mediated signaling, these agents reduce regulatory T cell proliferation and downregulate programmed death ligand 1 (PD-L1) expression, ultimately attenuating immunosuppressive responses [116]. Preclinical evidence demonstrates that combinatorial regimens incorporating β-blockers exhibit substantial anti-tumor activity; for instance, propranolol coupled with anti-angiogenic agents (e.g., bevacizumab) induces marked tumor regression in CRC xenograft models [117]. Notably, propranolol potentiates the effectiveness of immune checkpoint inhibitors via multifaceted mechanisms. It not only reduces tumor vascularization, enhances the infiltration of effector T cells, and decreases the accumulation of myeloid-derived suppressor cells, but also exhibits a synergistic effect when combined with anti-cytotoxic T-lymphocyte-associated antigen 4 (anti-CTLA4) treatment. Notably, the anti-tumor efficacy of propranolol relies on T cell activity, highlighting its pivotal function within neuro-immune regulation [118]. In contrast, parasympathetic activation supports the reestablishment of anti-tumor immune surveillance through the cholinergic anti-inflammatory pathway. As a non-invasive neuromodulatory method, vagus nerve stimulation increases parasympathetic tone and facilitates ACh secretion from cholinergic neurons. This signaling enhances natural killer cell cytotoxicity and reinstates the proliferative and cytotoxic functions of CD8⁺ T cells, thereby promoting immune equilibrium in TME [119].

## Challenges and prospects

This review systematically elucidates how the complex multidirectional dialogues between the central and peripheral components of the nervous system, the gut microbiota, and the immune system collectively drive the initiation, progression, and treatment resistance of CRC. This "neuro-tumor-immune" triad is a key driver of the malignant biological behavior of CRC. Based on these mechanisms, therapeutic strategies targeting neuro-tumor crosstalk—from traditional beta-blockers and neurotoxins to emerging inhibitors targeting specific neuropeptide receptors and neurotrophic factor receptors—offer promising new avenues for overcoming resistance to conventional therapies. In particular, combining these neurotargeted drugs with immune checkpoint inhibitors holds the potential to reverse immune therapy resistance by modulating the immunosuppressive tumor microenvironment. However, current preclinical models have inherent limitations in simulating the complex neural innervation and neuro-immune interactions of human tumors. Future research urgently needs to develop more advanced humanized models, such as using co-cultured organoids, neural chips, and genetically modified humanized mouse models to construct human tumor xenograft models with humanized immune systems and specific neural receptors, in order to better evaluate the efficacy of immune-neural interactions. In addition, there is a need to develop miniaturized biosensors for real-time monitoring of specific neurotransmitter concentration changes in the TME; identify predictive biomarkers; address drug delivery challenges; and conduct rational combination clinical trials to promote the clinical translation of this field. Finally, the potential consequences of targeting the nervous system must be carefully assessed. The comprehensive impact of long-term inhibition or activation of specific neural pathways on overall physiological function needs to be fully evaluated.

## Data Availability

No datasets were generated or analysed during the current study.

## Abbreviations

5-FU: 5-Fluorouracil
5-HT: Serotonin (5-Hydroxytryptamine)
ACh: Acetylcholine
ADRA2: Alpha-2 Adrenergic Receptor
ADRB2: Beta-2 Adrenergic Receptor
AhR: Aryl Hydrocarbon Receptor
ANS: Autonomic Nervous System
APC: Adenomatous Polyposis Coli
BDNF: Brain-Derived Neurotrophic Factor
BoNT: Botulinum Toxin
CAF: Cancer-Associated Fibroblast
cAMP: Cyclic Adenosine Monophosphate
CAP: Cholinergic Anti-inflammatory Pathway
CGRP: Calcitonin Gene-Related Peptide
CIAP2 (cIAP2): Cellular Inhibitor of Apoptosis Protein 2
CNS: Central Nervous System
CRC: Colorectal Cancer
CREB: cAMP Response Element-Binding Protein
CSC: Cancer Stem Cell
CTLA-4: Cytotoxic T-Lymphocyte-Associated Protein 4
DC: Dendritic Cell
DEX: Dexmedetomidine
DUSP4: Dual-Specificity Phosphatase 4
EC: Enterochromaffin Cell
EEC: Enteroendocrine Cell
EGC: Enteric Glial Cell
EGFR: Epidermal Growth Factor Receptor
EMT: Epithelial-Mesenchymal Transition
ENS: Enteric Nervous System
Epi: Epinephrine
ERK: Extracellular Signal-Regulated Kinase
FFAR2: Free Fatty Acid Receptor 2
FFAR3: Free Fatty Acid Receptor 3
Foxp3: Forkhead Box P3
GABA: Gamma-Aminobutyric Acid
GABABR: Gamma-Aminobutyric Acid Type B Receptor
GABABR1: Gamma-Aminobutyric Acid Type B Receptor Subunit 1
GAD: Glutamate Decarboxylase
GBA: Gut-Brain Axis
GDNF: Glial Cell-Derived Neurotrophic Factor
GDF15: Growth Differentiation Factor 15
GLP-1: Glucagon-Like Peptide-1
GLP-1R: Glucagon-Like Peptide-1 Receptor
GLP-2: Glucagon-Like Peptide-2
GLS: Glutaminase
GM-60186: HTR2B Antagonist GM-60186
GPCR: G Protein-Coupled Receptor
GPR43: G Protein-Coupled Receptor 43
GPR109A: G Protein-Coupled Receptor 109A
GZMB: Granzyme B
HIF-1α: Hypoxia-Inducible Factor 1-Alpha
HPA axis: Hypothalamic-Pituitary-Adrenal Axis
HTR1B: 5-Hydroxytryptamine Receptor 1B
HTR1D: 5-Hydroxytryptamine Receptor 1D
HTR1F: 5-Hydroxytryptamine Receptor 1F
HTR2B: 5-Hydroxytryptamine Receptor 2B
IBD: Inflammatory Bowel Disease
ICB: Immune Checkpoint Blockade
IBS: Irritable Bowel Syndrome
IDH3G: Isocitrate Dehydrogenase 3 Gamma
IL: Interleukin
JAG2: Jagged Canonical Notch Ligand 2
KRAS: Kirsten Rat Sarcoma Viral Oncogene Homolog
LDHA: Lactate Dehydrogenase A
M3R: M3 Muscarinic Acetylcholine Receptor
mAChR: Muscarinic Acetylcholine Receptor
MAPK: Mitogen-Activated Protein Kinase
MDSC: Myeloid-Derived Suppressor Cell
NE: Norepinephrine
NED: Neuroendocrine Differentiation
NF-κB: Nuclear Factor Kappa-B
NGF: Nerve Growth Factor
NK Cell: Natural Killer Cell
NMDA: N-Methyl-D-Aspartate
NMUR1: Neuromedin U Receptor 1
NMUR2: Neuromedin U Receptor 2
NTS: Nucleus Tractus Solitarius
PD-1: Programmed Cell Death Protein 1
PD-L1: Programmed Death-Ligand 1
PKA: Protein Kinase A
PNI: Perineural Invasion
PNS: Paraneoplastic Neurological Syndrome
PNS: Peripheral Nervous System
PVN: Paraventricular Nucleus
PYY: Peptide YY
ROS: Reactive Oxygen Species
SAM: Sympathetic–Adrenal–Medullary Axis
SC: Schwann Cell
SCFA: Short-Chain Fatty Acid
SLC22A3: Solute Carrier Family 22 Member 3
SP: Substance P
STAT3: Signal Transducer and Activator of Transcription 3
TAM: Tumor-Associated Macrophage
TCA Cycle: Tricarboxylic Acid Cycle
TET2: Ten-Eleven Translocation 2
TGF-β: Transforming Growth Factor-Beta
TGM2: Transglutaminase 2
Th17 Cell: T Helper 17 Cell
TLR4: Toll-Like Receptor 4
TME: Tumor Microenvironment
TNF-α: Tumor Necrosis Factor-Alpha
TP53: Tumor Protein P53
Treg: Regulatory T Cell
TrkA: Tropomyosin Receptor Kinase A
TrkB: Tropomyosin Receptor Kinase B
VEGF: Vascular Endothelial Growth Factor
YAP1: Yes-Associated Protein 1
ZEB1: Zinc Finger E-Box Binding Homeobox 1
β-AR: Beta-Adrenergic Receptor
